# Essential role of MED1 in the transcriptional regulation of ER-dependent oncogenic miRNAs in breast cancer

**DOI:** 10.1038/s41598-018-29546-9

**Published:** 2018-08-07

**Authors:** Neha Nagpal, Shivani Sharma, Sourobh Maji, Giorgio Durante, Manuela Ferracin, Jitendra K. Thakur, Ritu Kulshreshtha

**Affiliations:** 1 Department of Biochemical Engineering and Biotechnology, Indian Institute of Technology Delhi, New Delhi, 110016 India; 2 National Institute of Plant Genome Research, Aruna Asaf Ali Marg, New Delhi, 110067 India; 3 Department of Experimental, Diagnostic and Specialty Medicine (DIMES), University of Bologna, 40126 Bologna, Italy; 4 Present Address: Children’s Hospital, Harvard Medical School, Boston, MA USA

## Abstract

Mediator complex has been extensively shown to regulate the levels of several protein-coding genes; however, its role in the regulation of miRNAs in humans remains unstudied so far. Here we show that MED1, a Mediator subunit in the Middle module of Mediator complex, is overexpressed in breast cancer and is a negative prognostic factor. The levels of several miRNAs (miR-100-5p, -191-5p, -193b-3p, -205-5p, -326, -422a and -425-5p) were found to be regulated by MED1. MED1 induces miR-191/425 cluster in an estrogen receptor-alpha (ER-α) dependent manner. Occupancy of MED1 on estrogen response elements (EREs) upstream of miR-191/425 cluster is estrogen and ER-α-dependent and ER-α-induced expression of these miRNAs is MED1-dependent. MED1 mediates induction of cell proliferation and migration and the genes associated with it (JUN, FOS, EGFR, VEGF, MMP1, and ERBB4) in breast cancer, which is abrogated when used together with miR-191-inhibition. Additionally, we show that MED1 also regulates the levels of direct miR-191 target genes such as SATB1, CDK6 and BDNF. Overall, the results show that MED1/ER-α/miR-191 axis promotes breast cancer cell proliferation and migration and may serve as a novel target for therapy.

## Introduction

Mediator is an evolutionary conserved large multiprotein complex that plays critical role in relaying the regulatory signal from DNA-bound gene-specific transcription factors to the RNA polymerase II transcriptional machinery^1–3^. Mediator was discovered in yeast as a factor required for activator-dependent transcription of genes but now it is becoming clear that it is involved in almost every step of transcription of Class II genes, including pre-initiation complex formation, initiation of transcription or promoter clearing, transcript elongation, splicing of transcripts, gene looping and termination of transcription^4–9^. In metazoans, Mediator is composed of about 30 subunits arranged in four different modules viz Head, Middle, Tail and Kinase modules. It is considered that the Head, Middle and Tail modules form the core part of the complex whereas the Kinase module reversibly associates with it as and when required. The Kinase module is mainly involved in transcriptional repression by phosphorylating CTD (CTD- Carboxy-Terminal repeat Domain) heptads of RNA Pol II and some other proteins constituting the pre-initiation complex^10–12^. Being a gigantic complex, Mediator provides large and accommodating interface for several protein-protein interactions^13^. Generally, some specific subunits interact with specific DNA-bound transcription factors and other specific subunits interact with RNA Pol II and its accessory factors constituting transcriptional machinery, forming a communication bridge between the two. Consistent to this, Mediator is considered to be a ‘hub’ to integrate the signals from different transcription regulators and pass the ‘resultant’ signal to the transcriptional machinery^3^. RNA Pol II transcribes not only protein-coding genes but also the genomic regions that give rise to noncoding RNAs (ncRNAs). Role of Mediator in protein coding genes is extensively studied, but its importance in the regulation of ncRNAs is just emerging. In mouse embryonic stem cells, a stable molecular assembly formed between the Ada-Two-A-containing (ATAC) histone acetyltransferase and Mediator complex, regulates a subset of RNA pol II-transcribed unspliced ncRNA genes (mostly small nuclear RNA genes)^14^. In Arabidopsis, a set of Mediator subunits is involved in the transcription of ncRNAs that helps in recruitment of RNA pol V, a plant-specific polymerase evolved from Pol II^15^. In fission yeast, a triad of Med8-Med18-Med20 regulates transcription of ncRNA from centromeric region^16^. Thus, function of Mediator in the transcription of ncRNAs is emerging and seems to be conserved in eukaryotes.

MicroRNAs (miRNAs) are a family of well-studied small ncRNAs that regulate gene expression at post-transcriptional level by sequence-specific targeting of mRNAs causing translational repression or mRNA degradation^17,18^. There is hardly any report but one in plants, on regulation of miRNA expression by Mediator complex. In Arabidopsis, Mediator has been shown to be involved in miRNA biogenesis by recruiting RNA Pol II to the promoters of miRNA genes^15^. Although miRNA genes are also transcribed by RNA Pol II in metazoans^19^, the direct involvement of Mediator complex in the regulation of miRNA expression in metazoans has not been shown.

In this report, we show that MED1, a subunit in the Middle module of Mediator complex, is altered in upto 20% of breast cancer patients and is correlated to poor survival. MED1 is known to play an essential role in ER-α-mediated gene transcription^20,21^. We show that estrogen (E2)-activated ER-α targets MED1 to recruit Mediator complex to the promoter of miR-191/425 cluster inducing their transcription. Both miR-191 and MED1 promote breast cancer cell proliferation and migration. MED1-mediated increase in cell proliferation and migration is inhibited upon miR-191 downregulation, suggesting the presence of a MED1/ER-α/miR-191 oncogenic axis that could serve as an effective target for breast cancer therapy. MED1 is also shown here to be involved in the regulation of other breast cancer related miRNAs suggesting the crucial and yet unexplored role of Mediator complex in breast cancer pathogenesis through regulation of miRNAs.

## Results

### Deregulation of Mediator complex subunit 1 (MED1) in breast cancer patients

**Figure 1:**
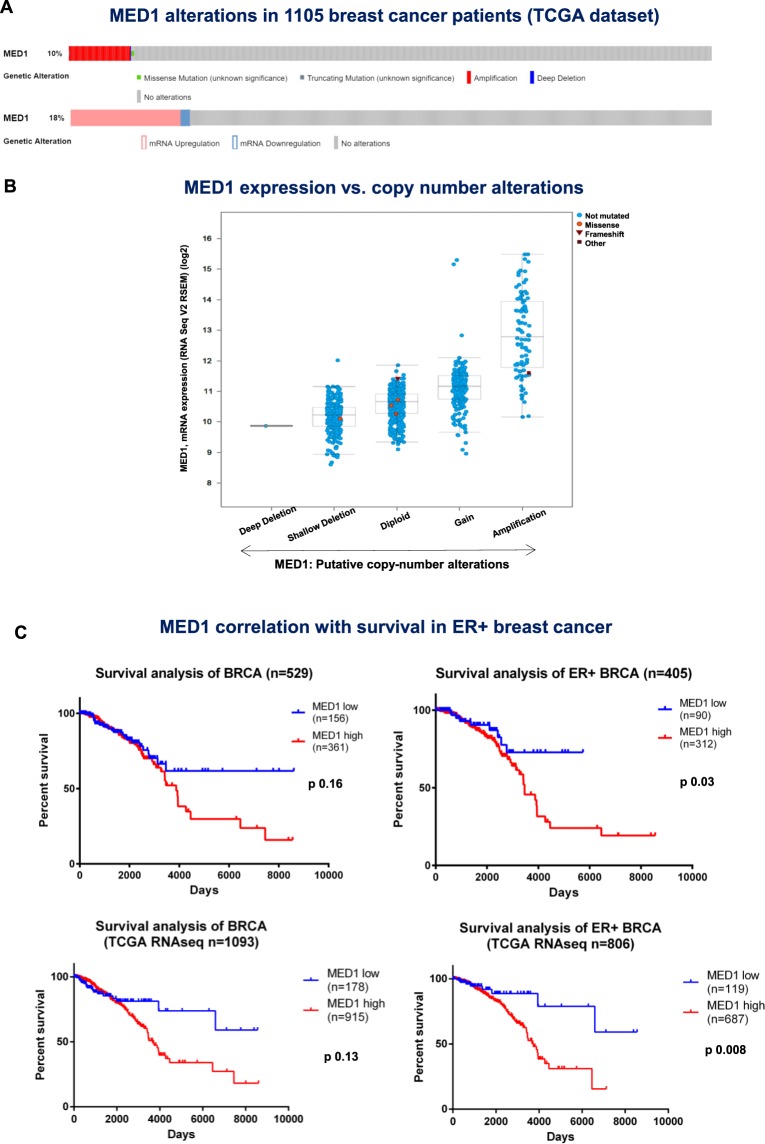
MED1 alterations in breast cancer patients. (**A**,**B**) Genetic and transcriptomic alterations of MED1 in breast cancer patients. MED1 copy number variations and expression alterations were found in breast cancer patients from TCGA database (**A**). Copy number variations of MED1 locus are correlated to variations in MED1 mRNA expression levels (**B**). (**C**) MED1 expression is significantly associated to survival in breast cancer patients. Survival data were obtained from TCGA data portal for two datasets of BC patients analysed with microarray (top graphs) or RNA sequencing (bottom graphs). Both datasets confirmed a significant positive association between MED1 expression and Overall Survival in ER+ BC patients.

We checked for MED1 alterations in breast cancer patients using TCGA database through cBioPortal website^22,23^. Analyzing 1105 invasive breast cancer cases for genetic alterations, we observed that MED 1 is amplified in 10% and mutated in 0.6% of BC patients (Fig. 1A). Upregulation of MED1 expression can be observed in ~20% of breast cancer patients (Fig. 1A). MED1 mRNA expression levels correlated with the copy number variations (Fig. 1B) and is significantly higher in ER (estrogen receptor)+ and ER- breast cancer compared to normal breast (Supplementary Data 1A,B). The association between MED1 transcript levels and prognosis of breast cancer patients was examined using TCGA dataset with clinical annotations. We found that breast cancer patients with higher MED1 levels had poor survival than patients with low MED1 levels with the results being more significant for the ER+ patients (Fig. 1C). Overall, these studies strongly indicated that MED1 is relevant in breast cancer and correlated with poor prognosis.

### MED1 regulates miRNA expression in breast cancer

**Figure 2:**
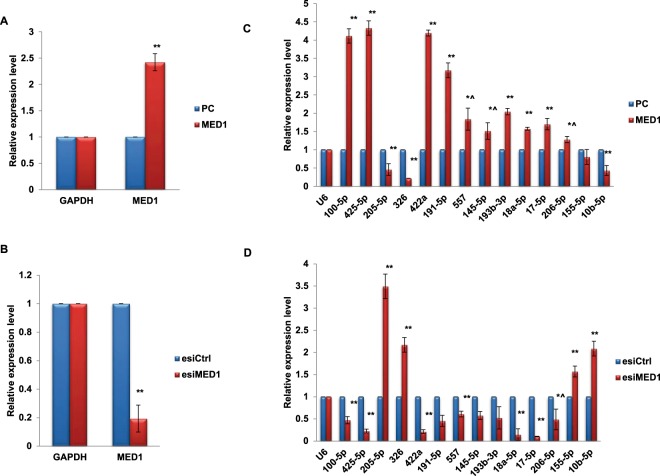
MED1 is involved in the regulation of miRNAs in breast cancer. Confirmation of overexpression/inhibition of MED1 transcript in MCF7 cells. MED1 was transiently up/downregulated by transfecting MCF7 cells with pCDNA3.1-MED1 (MED1) or pCDNA3.1 (PC) and esiMED1/esiCtrl. The corresponding effect on MED1 transcript levels in response to MED1 overexpression (**A**) or downregulation (**B**) was then checked by qRT-PCR and compared to that of their respective controls in MCF7 cells. (**C**,**D**) MED1 was overexpressed (**C**) or inhibited (**D**) in MCF7 cells and stem loop qRT-PCR was done to check the levels of various breast cancer related miRNAs. The mRNA data was normalized to GAPDH and miRNA data was normalized to U6 small RNA. The graphical data points represent mean + S.D of at least three independent experiments (**P<0.05, *P<0.1). Error bars denote + SD.

MED1 has been previously shown to regulate the level of various protein-coding genes in breast cancer^20,21^. However, its role in the regulation of miRNAs remains unstudied so far. Thus, we transiently modulated the levels of MED1 in MCF7 cells and checked for its effects on miRNA levels. Change in the expression level of MED1 in the transfected cells was confirmed by qRT-PCR and western blotting (Fig. 2A,B, Supplementary Data 2A). Next, we analysed the expression levels of miRNAs (miR-10b-5p, 17-5p, 18a-5p, 100-5p, 145-5p, 155-5p, 191-5p, 193b-3p, 205-5p, 206-5p, 326, 422a, 425-5p) that have been shown to be deregulated in breast cancer^24–28^. We found that the over-expression of MED1 brings about a significant induction in the levels of several of these miRNAs with the highest induction for miR-100-5p, miR-191-5p, miR-422a and miR-425-5p (Fig. 2C). We also observed downregulation of some of the miRNAs such as miR-10b-5p, -205-5p and -326 in response to MED1 overexpression (Fig. 2C). The regulation of these miRNAs by MED1 was further confirmed when opposite results were obtained upon MED1 inhibition (using esiRNA specific to MED1) (Fig. 2D, Supplementary Data 2A). Overall, the results strongly indicated that deregulation of MED1 affects the levels of several breast cancer associated miRNAs. Here in this study, we focused on MED1-mediated activation of a set of miRNAs known to be overexpressed in breast cancer.

### Interdependence of MED1 and ER-α to regulate miR-191/425 cluster

Next we wanted to know if MED1 is directly involved in the regulation of these miRNAs. Interestingly, we found a significant positive correlation between some of the highly induced miRNAs-(miR-191-5p, miR-425-5p, miR-100-5p, miR422a) and MED1 levels in breast cancer TCGA dataset (Supplementary Data 1C). We focused on the miRNA cluster, miR-191/425 which was found to be highly induced by MED1 overexpression and significantly downregulated by reduced levels of MED1 (Fig. 2C,D). The miR-191/425 cluster locus does not show any alteration (amplification/deletion) in breast cancer patients but the levels of miR-191 and miR-425 were found to be higher in breast cancer patients (Supplementary Data 3A–C). Several reports including ours have shown thatmiR-191 functions as an oncogenic miRNA in breast cancer by promoting cell proliferation and migration^24,25,29,30^. Also, both miR-191 and miR-425 are induced by estrogen (E2), a hormone implicated in breast cancer, through transcriptional regulation by ER^24,25^.

**Figure 3:**
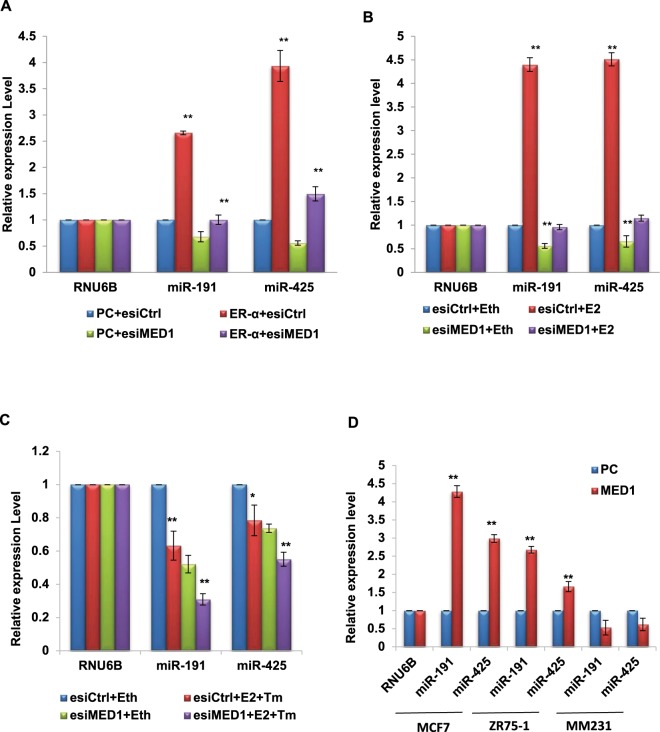
Effect of ER-α and MED1 on transcriptional regulation of miR-191/425 cluster. (**A–C**) MED1 or ER-α were overexpressed or inhibited and stem loop qRT-PCR was done to check the levels of miR-191 and miR-425 (**A**). Estrogen (E2) or vehicle (ethanol, Eth) treatment was given along with esiMED1 or tamoxifen (Tm) and the effect on miR-191/425 was observed using stem loop qRT-PCR. (**B**,**C**) The data was normalized to U6 small RNA (RNU6B) using 2^−ΔCt^ method. (**D**) Levels of MED1 were transiently modulated using pCDNA3.1-MED1 (MED1) or pCDNA3.1 (PC) in a panel of ER+ve/-ve breast cancer cell lines (MCF7, ZR75-1, MDA-MB-231) and effect on miR-191/425 levels was tested using stem-loop qRT PCR. Induced levels of both the cluster miRNAs (miR-191 and miR-425) were observed in response to MED1 overexpression in ER+ve cell lines (MCF7, ZR75-1) while opposite results were observed for ER-ve cell line (MDA-MB-231). Thereby confirming that MED1 mediated induction of miR-191/425 occurs in an ER-dependent manner. The graphical data points represent mean + S.D of at least three independent experiments (**P<0.05). Error bars denote + SD.

Recently, MED1 was also implicated in ER regulated growth of breast epithelial cells and breast carcinoma^31^. Many nuclear receptors including ER are known to target MED1 for their transcriptional response^32^. Therefore, we made an attempt to understand if ER collaborates with MED1 to regulate the expression of miR-191/425 cluster. For this, we overexpressed ER-α in MCF7 cells with/without MED1 inhibition and sought for the effect on miR-191/425 levels. We found that ER-α brings about induction of miR-191 and miR-425 levels in MCF7 cells, however, this increase cannot be observed when MED1 is silenced along with ER-α overexpression (Fig. 3A, Supplementary Data 2B). Similarly, estrogen treatment of MCF7 cells brought about induction of miR-191 and miR-425 levels. However, MCF7 cells treated with esiMED1 and/or tamoxifen were unable to induce miR-191 and miR-425 levels in response to estrogen treatment (Fig. 3B,C). This suggests that both ER-α and MED1 participate in the same pathway and their interaction might play a significant role in the transcriptional regulation of miR-191/425 cluster. To know if MED1 mediated induction of miR-191/425 is ER-dependent, we overexpressed MED1 in both ER+ve (MCF7, ZR-75) and ER-ve (MDA-MB-231) breast cancer cell lines and found that MED1 mediated induction of miR-191/425 is limited to ER+ve background (Fig. 3D). Next, we checked for the effect of MED1 overexpression/silencing on other ER-α regulated miRNAs. Interestingly, modulation of MED1 affected the levels of other ER-associated miRNAs (such as miR-10b, -100, -17, -18a, -205, -206, -326, -422a) as well suggesting that ER-α:MED1 mediated miRNA regulation may go beyond miR-191/425 cluster (refer Fig. 2C,D).

### ER-α recruits MED1 on the EREs upstream of miR-191/425 cluster to induce transcription

**Figure 4:**
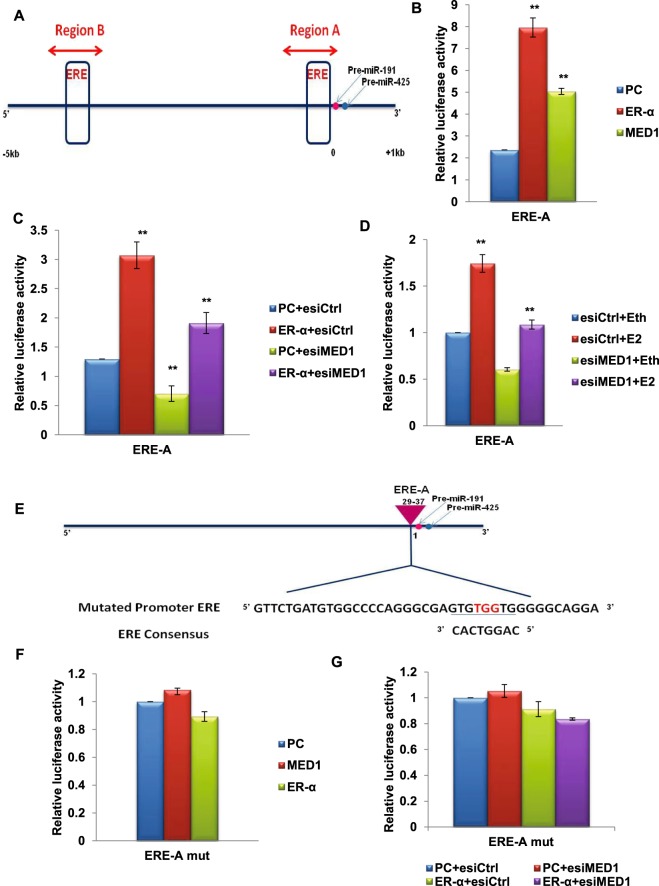
ERE present upstream of miR-191/425 cluster is responsive to ER-α or MED1 overexpression. (**A**) Diagram showing ERE consensus sequences in region upstream of pre-miR-191 using Promo 3.0 software. The locations of the two regions (ERE-A and ERE-B) used for luciferase promoter assays are marked. (**B**) Graph showing luciferase activity of ERE-A luciferase construct of miR-191/425 cluster in response to ER-α or MED1 overexpression. (**C**,**D**) Estrogen or ER-α mediated effect on luciferase activity of ERE-A construct when coupled with MED1 inhibition. Levels of MED1 were modulated (using esiMED1/esiCtrl) with/without ER-α overexpression or estrogen stimulation (10^−9^ M E2; estrogen or 10^−9^ M Eth; ethanol) and effect on luciferase activity of ERE-A (luciferase constructs of miR-191/425 cluster) was observed. Increase in luciferase activity in response to ER-α (**C**) or estrogen treatment (**D**) was not observed when estrogen stimulation was given along with inhibition of MED1. (**E**) Diagram showing wild type/mutated ERE-A region upstream of miR-191/425 cluster. (**F**,**G**) Graph showing promoter luciferase activity of ERE-A mut (luciferase constructs bearing mutated ERE site), in response to differential expression of ER-α and MED1 alone (**F**) or in combination (**G**). The graphical data points represent mean + S.D of at least three independent experiments (**P<0.05). Error bars denote + SD.

**Figure 5:**
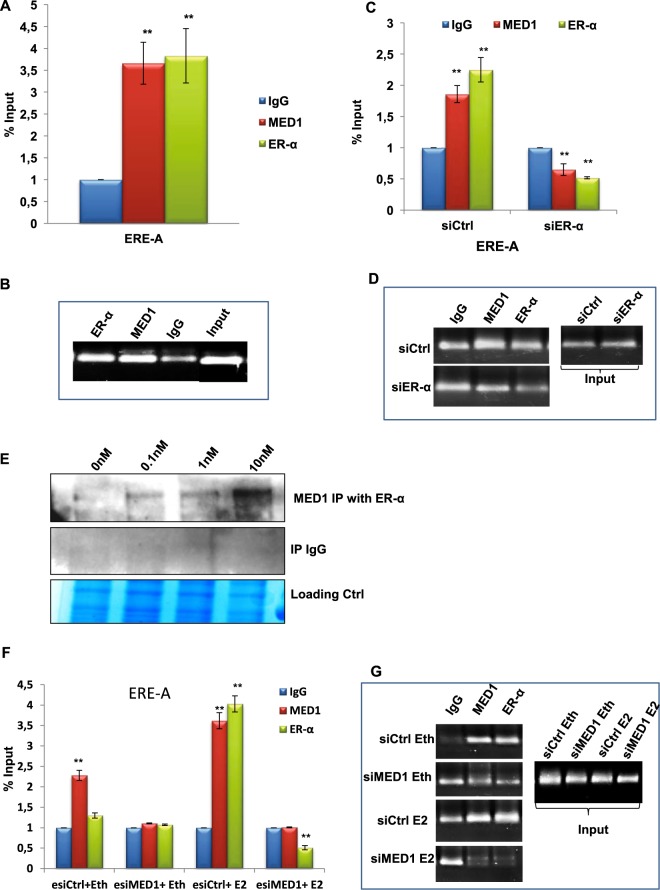
Recruitment of MED1/ER-α on the miR-191/425 promoter ERE. (**A**,**B**) qPCR (**A**) or semi-quantitaive (**B**) data of CHIP assay showing % input of bound chromatin on ERE-A element using antibody specific to MED1/ER-α. (**C**,**D**) qPCR (**C**) or semi-quantitative (**D**) data of CHIP assay shows recruitment of both ER-α and MED1 on ERE-A in response to ER-α inhibition using specific siRNA. (**E**) Estrogen dependent MED1 and ER-α interaction in MCF7 cells. MCF7 cells were starved for estrogen for 3-4 days followed by estrogen treatment (0–10 nM) for 45 min. The cells were lysed and probed for the interaction of ER-α and MED1 using co-immunoprecipitation assay. ER-α antibody was used for pull down and western blotting was done with MED1 specific antibody. (**F**,**G**) CHIP assay was performed using ER-α or MED1 antibodies in response to 10^−8^ M estrogen (E2) or ethanol (Eth) treatment for 2 hrs with/without inhibition of MED1. qPCR (**F**) or semi-quantitative (**G**) data shows PCR based quantification of bound chromatin (% input) on ERE-A (element present upstream of miR-191/425 cluster) in MCF7 cells. (**P<0.05). Error bars denote + SD.

ERs are known to function by binding to the estrogen responsive elements (EREs) present in the regulatory region of the target genes. Our previous study demonstrated the recruitment of ERs on the EREs present in region A (27bp upstream of miR-191) and region B (~4.2 kb upstream of miR-191) in response to estrogen treatment^24^ (Fig. 4A). Thus, we were interested to know if MED1 also requires ERE for its effect on miR-191/425 expression. To address this question, we conducted dual promoter luciferase assay on cloned EREs as mentioned in the materials and methods. Similar to ER-α, over-expression of MED1 induced luciferase activities driven by either ERE-A or ERE-B (Fig. 4B, Supplementary Data 4A). Importantly, inhibition of MED1 reduced ER-α-induced luciferase activity under the control of either of the EREs, A or B (Fig. 4C, Supplementary Data 4B). Similar results were obtained when estrogen treatment was given instead of ER-α overexpression. Estrogen stimulation enhanced luciferase activity while no change was observed when estrogen stimulation is combined with MED1 inhibition (Fig. 4D). Thus, both ER-α and MED1 act on the same binding sites (EREs) present in the regulatory region of miR-191/425 cluster. This was further confirmed when mutation was introduced in the ERE-A at the site of ER-α binding consensus and no change in luciferase activity was observed with either overexpression of MED1 or ER-α (Fig. 4E–G). In order to confirm the recruitment of MED1 on these EREs, chromatin immunoprecipitation (ChIP) was performed using the antibodies specific to human MED1 (rabbit IgG was used as control) in MCF7 cells. The results revealed that similar to ER-α, MED1 is also recruited to the EREs (A and B) upstream of miR-191/425 cluster (Fig. 5A,B Supplementary Data 4C). To know if ER-α is involved in the recruitment of MED1 on the EREs, we checked for MED1 binding to EREs in presence of ER-α siRNA (ER-α inhibition). Interestingly, recruitment of MED1 on these EREs was severely inhibited in the absence of ER-α (Fig. 5C,D, Supplementary Data 4D). Altogether, we show that recruitment of MED1 to the promoter of miR-191/425 cluster is ER-α dependent. Our results also confirm that ER-α requires MED1 to induce the transcription of miR-191/425 cluster.

### Estrogen enhances the physical interaction between ER-α and MED1

MED1 has been reported to physically interact with several nuclear receptors including ERs, and help them in inducing the transcription of their target genes^31,32^. Since recruitment of MED1 to the EREs present in the promoter region of miR-191/425 cluster is ER-α dependent and ER-α-regulated transcription of this cluster is MED1 dependent, we sought to study the interaction between ER-α and MED1. To address this, we performed co-immunoprecipitation experiment in the presence of increasing dose of estrogen. Interestingly, we found that estrogen enhances the physical interaction between ER-α and MED1 (Fig. 5E). Further, ChIP experiment was performed to investigate the occupancy of ER-α and MED1 on EREs in the presence of estrogen. Estrogen treatment induced the occupancy of both ER-α and MED1 on the EREs, suggesting that recruitment of ERs and MED1 on EREs is estrogen dependent (Fig. 5F,G, Supplementary Data 4E). Overall, these results show that in presence of estrogen, ER-α recruits MED1 to the EREs upstream of miR-191/425 cluster to enhance their transcription.

### Functional implications of MED1-miR-191 axis in breast cancer

**Figure 6:**
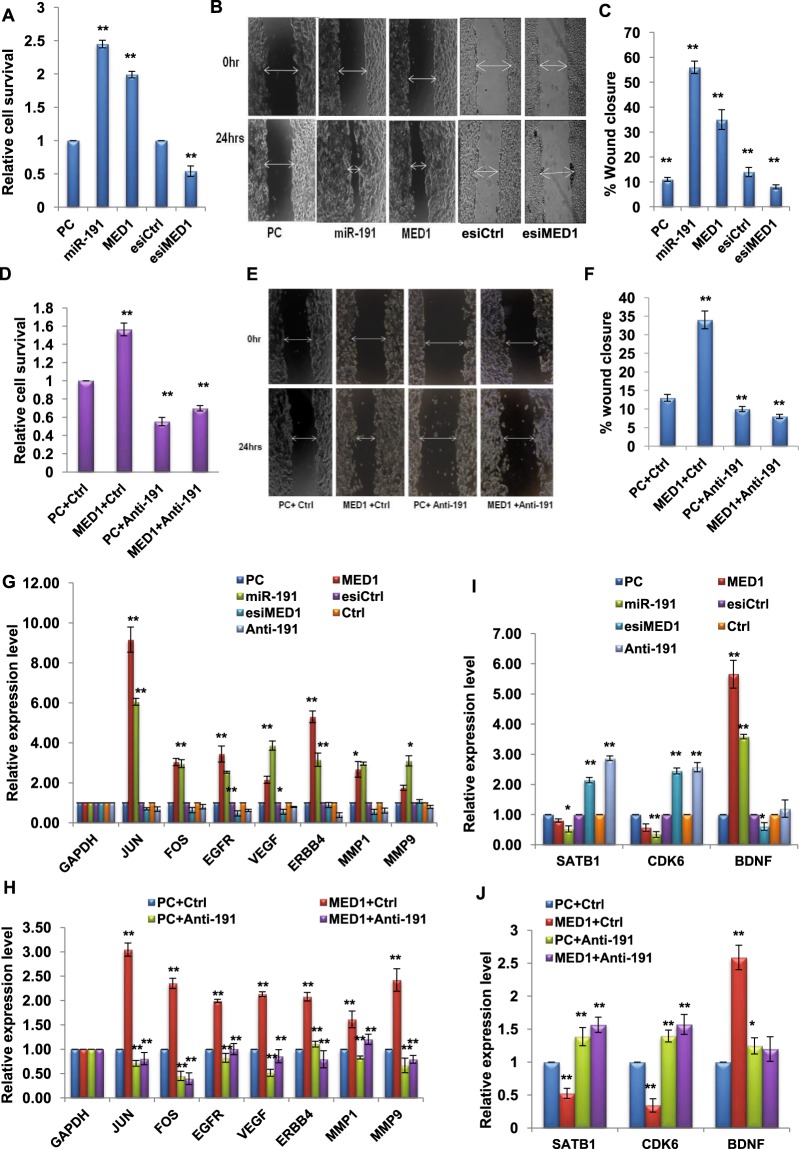
miR-191 acts as the downstream effecter of MED1 mediated cellular functions. (**A–C**) Functional implications of MED1 or miR-191 overexpression in breast cancer. MCF7 cells were transfected with MED1 or miR-191 or PC (control vector) and MTT assay was performed (48 hrs post transfection) to measure effect on cell survival. The results show that both MED1 and miR-191 promote cell survival (**A**). (**B**,**C**) Wound healing assay was performed to observe the effect of miR-191 and MED1 on cell migration. MCF7 cells were transfected with MED1 or miR-191 or control vector and gap or wound closure was observed (**B**) using Nikon microscope, 10X magnification and % gap closure was quantified using arbitrary scale (**C**). Graph shows that more healing (gap closure) was observed in response to both miR-191 and MED1 overexpression, hence both MED1 and miR-191 impart enhanced cell migratory capacity to the cells. (**D–F**) MED1 mediated cellular effects are miR-191 dependent. MCF7 cells were transiently transfected with MED1/PC (vector control) along with inhibition of miR-191(anti-miR-191)/Ctrl and effect on cell survival was looked for using MTT assay (**D**). Increase in cell survival due to MED1 overexpression was significantly reduced when miR-191 was inhibited along with MED1 overexpression (**E**). Wound healing assay was performed to confirm the effect of MED1 and miR-191 on cell migration. Levels of MED1 were transiently overexpressed along with/without miR-191 inhibition (using anti-miR-191/ctrl) and gap closure was observed after 24 hrs. Comparatively, lesser gap was filled when MED1 overexpression was coupled with miR-191 inhibition (**F**) when quantified using arbitrary scale thereby, confirming that miR-191 is a downstream effecter for MED1 mediated cellular migration. (**G**) qRT-PCR data showing transcript levels of JUN, FOS, EGFR, VEGF, MMP1 and ERBB4 on miR-191 or MED1 overexpression/inhibition. GAPDH has been used for normalization of qRT-PCR data. (**H**) qRT-PCR data showing transcript levels of JUN, FOS, EGFR, VEGF, MMP1 and ERBB4 on MED1 overexpression or miR-191 inhibition. (**I**) qRT-PCR data showing transcript levels of established miR-191-target genes (SATB1, CDK6 and BDNF) in response to both miR-191 or MED1 overexpression/inhibition. GAPDH has been used for normalization of qRT-PCR data. (**J**) qRT-PCR data showing transcript levels of established miR-191-target genes (SATB1, CDK6 and BDNF) on MED1 overexpression or miR-191 inhibition. (**P<0.05, (**P<0.05, * P<0.1). Error bars denote + SD.

We next looked for functional consequences of MED1-miR-191 connection in context of breast cancer. For this, we transiently modulated the levels of MED1 and miR-191 in a breast cancer cell line, MCF7 and checked for its effects on cell survival using MTT assay. We found that overexpression of both MED1 and miR-191 promoted cell survival in MCF7 cells (Fig. 6A). Similarly, increased cell migration was observed in wound healing assay in response to both MED1 and miR-191 overexpression in MCF7 cells (Fig. 6B,C). In contrast, inhibition of MED1 (using MED1 esiRNA) brought about inhibition of cell survival and migration (Fig. 6A–C).

Next, we looked for the functional impact of MED1-mediated regulation of miR-191 on breast cancer cell survival and migration. miR-191 inhibition oligos (anti-miR-191) along with its control were transfected with/without MED1 overexpression and effect on cell survival was observed (Fig. 6D). MED1-mediated increase in cell survival was reduced when MED1 was overexpressed along with anti-miR-191. Similar effects were obtained on MED1-mediated cellular migration (Fig. 6D–F).

In line with these observations, increase in the expression of genes involved in breast cancer cell proliferation or migration (JUN, FOS, EGFR, VEGF, MMP1 and ERBB4) was observed in response to both miR-191overexpression and MED1 overexpression (Fig. 6G). We found that MED1 mediated increase in the levels of genes involved in breast cancer migration or proliferation was inhibited when anti-miR-191 treatment was given along with MED1 overexpression (Fig. 6H). This suggests that MED1-mediated cellular effects are partly mediated through miR-191 thus, indicating miR-191 to be a downstream effect or of MED1 in breast cancer.

We also looked at the effects of MED1 on miR-191 direct cellular targets^24^ (SATB1, CDK6 and BDNF). Notably, both MED1 and miR-191 showed similar effects on these genes further suggesting the existence of MED1-miR-191 axis (Fig. 6I,J).

## Discussion

Mediator is a huge complex made up of almost 30 subunits in human. Mutational and genetic analysis in yeast, animals and plants suggest that to a certain extent, different subunits of Mediator are involved in regulating different sets of genes^3^. It seems that the essential subunits are required for transcription of virtually all protein coding genes and nonessential subunits have specialized, gene-selective roles in transcription initiation and its regulation. Though MED1 is a nonessential subunit in yeast, in last one decade, it has emerged as a pivotal component of the mammalian Mediator complex. MED1 deletion is embryonic lethal at midgestation confirming that it is an essential cofactor for proper embryonic development^33^. Mediator isolated from MED1 knockout cell lines is stable and transcriptionally active suggesting that MED1 is not important for the integrity of the complex^34,35^. MED1 is a very common target for nuclear receptors including ER. It has been shown that ectopic expression of MED1 significantly enhances ER-α functions and its silencing impairs ER-α-regulated transcription and estrogen-dependent proliferation of breast cancer cells^20,21,35^.

Based on TCGA data analyses of a dataset with >1000 breast cancer patients, MED1 was found to be altered (majorly amplifications) in upto 20% of these patients and its transcript levels correlated well with the copy number variations^22,23^. A previous study reported MED1 overexpression in ~50% of primary breast cancers (n = 15) and breast cancer cell lines (n = 6)^36^. Here we have shown that MED1 promotes breast cancer cell proliferation and migration. The levels of several proliferation and migration associated genes were induced upon MED1 overexpression. Based on analyses of various datasets using TCGA clinical data, high MED1 levels were found to correlate with poor prognosis of breast cancer patients^22,23^. Additionally, recent studies demonstrated that MED1 knockdown makes breast cancer cells more sensitive to anti-estrogen fulvestrant or tamoxifen treatment^37,38^. Overall, MED1 is involved in human breast carcinogenesis and treatment response and thus, understanding its functioning is imperative. Our results here identified MED1 as a regulator of several miRNAs known to be involved in breast cancer such as miR-10b-5p, -100-5p, -17-5p, 18a-5p, -191-5p, 193b-3p, 205-5p, -326, -422a and -425 suggesting its importance in breast cancer pathogenesis. MED1 was also shown to have significant positive correlation with the levels of miR-191-5p, miR-425-5p, miR-422a and miR-100-5p in breast cancer patients. There are several reports that suggest that Mediator complex plays an important role in both activation and repression of RNA pol II-mediated transcription of genes. For instance, MED14, which like MED1 is also targeted by several nuclear factors has been found to repress transcription of several genes in yeast and plants^39,40^. Similarly, MED15 and MED16 are known to be involved in both positive and negative regulation of transcription^39,41^. It is suggested that transcriptional regulation by Mediator subunits depends on the structure of the promoter, combination of *cis* elements and overall chromatin organization in the context specific manner^39,41–43^. In accordance with this, human Mediator has been found to act as both an activator and repressor, depending on the conditions used in the cell free transcription assay^44–46^. Interestingly, we noticed that some of the miRNAs that have been reported to show positive correlation with ER levels such as miR-100, -17, -18a,-191, -422a and miR-425 were MED1 induced while those known to show negative correlation with ER levels such as miR-10b and miR-155 showed downregulation by MED1 with exceptions such as miR-326, known to be induced by ER but downregulated by MED1^24–28^. An interesting negative feedback loop was also identified here since a miRNA known to target MED1^47^, miR-205, was found to be downregulated by MED1. It is also likely that MED1 mediated regulation of all miRNAs is not limited to estrogen receptor context. MED1 is known to interact with several transcription factors such as Thyroid hormone receptor, Androgen receptor, Glucocorticoid receptor, BRCA1, Hepatocyte nuclear factor 4 alpha, Peroxisome proliferator-activated receptor gamma, p53 etc^32–35,48,49^ and thus, MED1 mediated regulation of specific miRNAs may be guided by its interactions with various transcription factors. Besides, whether all the miRNAs are direct MED1 targets or not has not been investigated yet for any miRNA except miR-191 and miR-425. It may be that some miRNAs are getting regulated due to indirect regulation of its activators or repressors by MED1.

**Figure 7:**
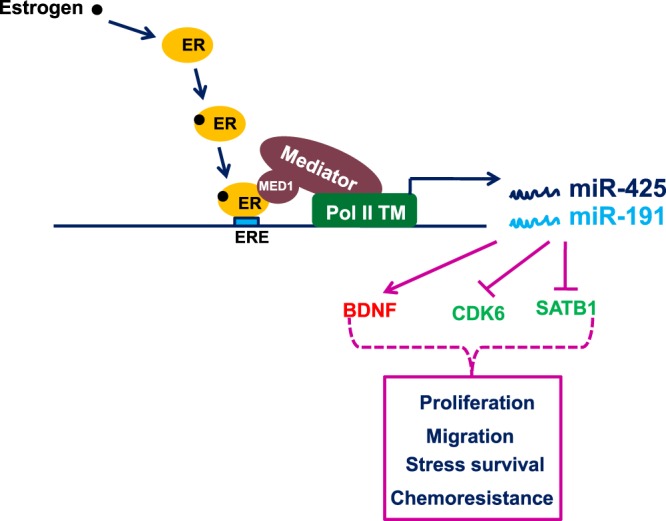
A proposed model detailing interplay of ER-α-MED1-miR-191/425 in breast cancer. A model for estrogen/ER-α induced MED1 mediated miR-191/425 regulation in breast cancer.

We focused on the oncogenic miRNA cluster, miR-191/425, that have previously been shown to be estrogen/ER regulated miRNAs that promote cell proliferation, migration and chemoresistance in ER positive breast cancer^24,25,29,30^. Our results here show that estrogen induced transcription of miR-191/425 is also MED1-dependent suggesting commonality in the two in terms of their target promoters. Indeed, ER-α and MED1 occupy same EREs in the regulatory region of miR-191/425 cluster. Furthermore, occupancy of MED1 on these EREs is ER-α-dependent indicating interaction between the two is imperative for miR-191/425 cluster regulation. This study reveals that interaction of ER-α and MED1 is ligand-dependent since binding of estrogen to ER increases occupancy of both ER-α and MED1 on the EREs present in the upstream region of miR-191/425 cluster. It is well established that Mediator stabilizes and facilitates pre-initiation complex (PIC) formation^50,51^. On the basis of these results and earlier reports, we propose a working model of estrogen induced expression of miR-191 and miR-425 (Fig. 7). Binding of estrogen to ER-α changes the conformation of ER-α in a way that it can interact with MED1. The estrogen-ER-α-MED1 complex bound to EREs, brings Mediator complex and RNA pol II transcriptional machinery to the promoter of miR-191/425 cluster and forms the PIC to induce the transcription of miR-191 and miR-425. Since our results show that the expression of other ER-regulated miRNAs (such as miR-10b, -100, -17, 18a, -191, 205, -206, -326, -422a and -425) is also affected by modulation of MED1 levels, we presume that MED1:ER-α association is working through the similar mechanism in context of other miRNAs as well. MED1 is also known to act as a cofactor for P53, GATA2, MYC and other cancer related proteins, but whether these associations are involved in regulation of ncRNAs is not yet known. Overall, it seems that MED1 overexpression leading to deregulation of protein coding genes and miRNAs in breast cancer may have far more consequences than previously thought and may go beyond breast cancer as well.

We show that both MED1 and miR-191 promote cell proliferation and migration in breast cancer and induce the expression of several genes associated with these properties. The functional link between MED1 and miR-191 is demonstrated since MED1 mediated induction of these properties is abrogated when MED1 is overexpressed along with miR-191 inhibition. We had previously identified a set of direct targets (SATB1, CDK6 and BDNF) of miR-191 in breast cancer^24^. Interestingly, miR-191 targets were found to be co-ordinately regulated by MED1 also supporting the existence of MED1-miR-191 axis in breast cancer.

In a miRNA screen with human placental trophoblasts under hypoxic conditions, MED1 was identified as a target of miR-205^47^. MED13, a subunit in the Kinase module is targeted by miR-378 and miR-208a to regulate mitochondrial metabolism, insulin sensitivity and glucose tolerance in mice^52,53^. Thus, in animals it seems that miRNAs play important role in regulating Mediator subunit composition and so its activity. However, role of Mediator or its subunits in the regulation of miRNAs in animals is not yet discovered. Our study reveals the involvement of a Mediator subunit, MED1, in the regulation of expression of miRNA genes, miR-191 and miR-425, in humans and provides the mechanism for it. Recently, in Arabidopsis, a dicot plant, three Mediator subunits were implicated in the transcription of few miRNA genes^14^. This is interesting because unlike metazoans, plant genomes do not code for nuclear receptors and MED1, which we found to be the main regulator of miRNA gene transcription in this study. However, plants do have their own set of plant specific TFs. More than 40 percent of TFs found in Arabidopsis and rice are specific for plants. Thus, though different sets of TFs and Mediator subunits are involved in transcription of miRNA genes in animals and plants, involvement of Mediator in miRNAs regulation seems to be conserved in two major eukaryotic kingdoms suggesting that this mechanism of gene regulation evolved before the diversification of plantae and animalia.

## Materials and Methods

### Cell culture

The human breast cancer cell line, MCF7, was a kind gift from Dr. Mircea Ivan (Indianapolis University, source ATCC). MDA-MB-231 and ZR-75 cells were purchased from NCCS cell line repository, Pune. Cells were maintained in RPMI 1640 (GIBCO) medium supplemented with 100 U/ml penicillin, 100 µg/ml streptomycin and 10% fetal bovine serum and incubated at 37 °C in 5% CO_2_ in incubator (Shell labs).

### Transient Transfections

Cells were seeded (5 × 10^5^ cells/well) in 6-well plates. In case of plasmids (ER-α, MED1 and their control vector pCDNA3.1), 2.5 µg of plasmid was transfected into 6-well plates using Lipofectamine 2000 (Invitrogen). In case of siRNAs (siER-α & siCtrl; Sigma), 100 nM while in case of esiRNAs (esiMED1 & esiCtrl; Sigma), 2 µg/µl (final concentration) were transfected using Lipofectamine 2000. After 6 hrs, media was changed and the samples were assayed after 48 hrs for miRNA levels (through stem-loop RT-PCR) or other cellular assays. Each experiment was repeated three times.

### RNA isolation and Stem-loop qRT-PCR

Total RNA isolation from cell lines was done using RNA Isolation kit (GeneJET RNA purification kit, Thermo Scientific), according to manufacturer’s instructions. Stem-loop RT-PCR was done to determine the level of all the miRNAs and RNU6B (used for normalization) in all the samples. 500 ng of total RNA was used for cDNA formation using RevertAid first strand cDNA synthesis kit (Fermentas). Specific RT primers are used for each miRNAs:

miR-191RT- GTCGTATCCAGTGCAGGGTCCGAGGTATTCGCACTGGATACGACCAGCTG, RNU6BRT-GTCGTATCCAGTGCAGGGTCCGAGGTATTCGCACTGGATACGACAAAATATGGAAC, miR-425RT- GTCGTATCCAGTGCAGGGTCCGAGGTATTCGCACTGGATACGACTCAACG, miR- 100RT- GTCGTATCCAGTGCAGGGTCCGAGGTATTCGCACTGGATACGACCACAAG, miR-205RT- GTCGTATCCAGTGCAGGGTCCGAGGTATTCGCACTGGATACGACCAGACTC, miR-326RT- GTCGTATCCAGTGCAGGGTCCGAGGTATTCGCACTGGATACGACCTGGAG, miR-422aRT- GTCGTATCCAGTGCAGGGTCCGAGGTATTCGCACTGGATACGACGCCTTC, miR-557RT- GTCGTATCCAGTGCAGGGTCCGAGGTATTCGCACTGGATACGACTTCAGT, miR-17RT- GTCGTATCCAGTGCAGGGTCCGAGGTATTCGCACTGGATACGACCTACCT, miR-18aRT- GTCGTATCCAGTGCAGGGTCCGAGGTATTCGCACTGGATACGACCTACCT, miR-206RT- GTCGTATCCAGTGCAGGGTCCGAGGTATTCGCACTGGATACGACCCACAC, miR-193bRT- GTCGTATCCAGTGCAGGGTCCGAGGTATTCGCACTGGATACGACAGCGGGA, miR-145RT- GTCGTATCCAGTGCAGGGTCCGAGGTATTCGCACTGGATACGACAGGGAT, miR-10bRT- GTCGTATCCAGTGCAGGGTCCGAGGTATTCGCACTGGATACGACCACAAA. Taqman miRNA probe, Assay ID-Hs03910067_s1 (Ambion) was used for miR-155.

qPCR was performed using miRNA specific forward and a common Stem-loop reverse universal primer (Reverse Primer- GTGCAGGGTCCGAGGT). The sequence of forward primers used are: miR-191F- CGCGCAACGGAATCCCA, miR-425F- GCCGAATGACACGATCACTCC, RNU6BF- GCCCCTGCGCAAGGATGAC, miR-100F- GCAACCCGTAGATCCG, miR-205F- GCTCCTTCATTCCACCG, miR-326F- GATACCTCTGGGCCCTTC, miR-422aF- GCGCGCACTGGACTTAGGGTC, miR-557F- GCGCGCTACAGTACTGTG, miR-17F- GCGCGCAAAGTGCTTACAGTG, miR-18aF- GCGCGTAAGGTGCATCTAGTGC,miR-206F- GGAGTAGTGGAATGTAAGGAAGT, miR-193bF- GTCAACTGGCCCTCAAAG, miR-145F- AGACGGCAGGTCAGGTCCAC, miR-10bF- GCGTACCCTGTAGAACCG, GAPDHF- GTCCATGCCATCACTGCCAC.

For gene transcript level quantification, the primers used are: GAPDHR- AGACGGCAGGTCAGGTCCAC, MED1F- CGAGTTAGGATCTGGATGAAGG, MED1R- GAAGTTGACTTCATGTCCTTGC, JUNF- GTGTGCACGAGTGGGAAGG, JUNR- GATCGAATGTTAGGTCCATGGAG, FOSF- AATGACCCTGAGCCCAAGCC, FOSR- AGCTCTGTGGCCATGGCC, VEGFF- GCTACTGCCATCCAATCGAGAC, VEGFR- CTATGTGCTGGCCTTGGTGAG, SATB1F- TCAGCAACAGCAGCAGCAAC, SATB1R- GATTCCCAAGGCTTCCACTG, CDK6F- GTGTGGAAATTCACTGCCTGG, CDK6R- TCAGAGAGCTGTGCTGCACC, BDNFF- AACTCAGGCCGAATGATCAAG, BDNFR- AAATGGCAGAGGTGAGGCAG, EGFRF- TGAGCAAGGAGCACAAGCCAC, EGFRR- AGAATTCCATCCCCTCCGTTTC, MMP1F- AGAGCAGATGTGGACCATGCC, MMP1R- TGAGCATCCCCTCCAATACCTG, ERBB4F- TCTCCCTGGCTGGTGTGTCTC, ERBB4R- TCCCTCTCTCACCCAAACTGATG.

### Promoter-Luciferase constructs

For promoter analysis, we used the promoter fragments (ERE-A and ERE-B) that encompass estrogen responsive elements present upstream of miR-191 and miR-425 promoter fragments. 2 EREs upstream of miR-191, with 100% match with ER-α consensus were cloned downstream of a luciferase promoter vector PGL3-tk-luciferase, (Promega) as previously described in Nagpal *et al*.^24^. The estrogen response element-A (ERE-A), the binding site for ER-α and MED1 in the upstream region of miR-191/425 cluster was mutated through site-directed mutagenesis with primers spanning the ER-α/MED1 binding site with indicated mutations (refer Fig. 4E) using inverse PCR and luciferase activity was measured. The sequences of primers used for cloning and site-directed mutagenesis are given below:

ERE-A-cloning-F- ATTCTCGAGCAGCTGCTTTTGGGATTCCG

ERE-A-cloning-R- ATAACGCGTCACCAGGGAAGCTCAACGG

ERE-A-mutF- AGGGCGAGTGTGGTGGGGGCAGGAGCTCC

ERE-A-mutR- GGAGCTCCTGCCCCCACCACACTCGCCCT

ERE-B-cloning-F- ATACTCGAGGCATGAGGTATGGCAGAGG

ERE-B-cloning-R- ATAACGCGTACCACTGCCCTTATCTTGCCTG

### Dual Luciferase assay

For luciferase assays, MCF7 cells (10000 cells/well) were cotransfected with 500 ng/well of each construct ERE-A and ERE-B along with ER-α/MED1/parent vector (PC) using Lipofectamine 2000 (Invitrogen). To confirm role of MED1 in binding of ER-α at these promoter fragments, 500 ng/well of each construct was cotransfected along with 500 ng each of ER-α/parent vector (PC) and 2 µg/µl of inhibitor for MED1; esiMED1/esiCtrl using Lipofectamine 2000 (Invitrogen). pRL-TK (10 ng/well) was transfected in all the wells for normalization of transfection efficiency. The activities of Firefly (Photinus pyralis) and Renilla (Renilla reniformis) luciferase were quantified with the Dual Luciferase Reporter Assay (Promega) 48 hrs post-transfection.

### Western Blot

Cells were transfected with esiMED1/esiCtrl and 48 hrs post-transfection, cells were lysed. 150–200 µl (per well of a 6-well) of RIPA buffer was used for cell lysis and harvested lysate was stored at −80 °C till further use. The protein concentration was determined by using Bradford Reagent (Sigma Aldrich). Equal amount of protein lysates were separated with 8–10% SDS-PAGE and transferred to nitrocellulose membrane (Amersham Hybond ECL). The membrane was then probed with a specific primary antibody at a dilution of 1:2000 (MED1, Santa Cruz Biotechnology) followed by washing and incubation with secondary antibody (anti-goat, HRP-linked; MED1). The specific protein band was visualized by autoradiography using an ECL kit (Amersham ECL Prime). The quantification of band density was then done by using GelQuantNET software (Biochem lab solutions).

### Chromatin Immunoprecipitation

To confirm recruitment of MED1 to the promoter of miR-191/425 cluster, 70–80% confluent MCF7 cells were crosslinked, washed, and resuspended in RIPA buffer as described above. For ER-α mediated recruitment of MED1, cells were transfected with siRNA for ER-α or siCtrl and 48 hrs post transfection cells were crosslinked and used for CHIP analysis. For ligand-dependent effect of MED1, MCF7 cells were starved for estrogen (cultured in phenol red free medium with dextran-stripped serum for 3 days) and tranfected with esiMED1/esiCtrl. 48 hrs post transfection, estrogen treatment (1 × 10^−8^ M) was given for 2 hrs and cells were then crosslinked, washed and resuspended in RIPA buffer for CHIP. To confirm the interaction of MED1 and ER, MCF7 cells were starved for estrogen for 3–4 days (as described above) and estrogen treatment (0–10 nM) was given for 45 min. followed by cell crosslinking and lysis using RIPA buffer. Immunoprecipitation was performed as previously described in Nagpal *et al*.^24^. Briefly, cells were crosslinked, washed and lysed followed by sonication. Specific antibodies against ER-α/MED1 (Santa Cruz Biotechnology), salmon sperm DNA (Sigma) and pre-equilibrated protein A-sepharose beads (30 µl) were added to the chromatin extract and incubated overnight, washed and eluted with 0.5%w/v SDS solution. For protein-protein interaction (ER-MED1), the immunoprecipitated proteins were eluted in SDS loading buffer and are separated by using SDS-PAGE gel (8%) followed by western detection using MED1-specific was used as control antibody while the chromatin extract without any antibody/beads treatment was used as positive control. For DNA sequence specific quantification qPCR was done. For chromatin immunoprecipitation, the precipitated chromatin extract was decrosslinked at 65 °C for 4–5 hrs and DNA was then purified with PCR purification kit (HiMedia). Rabbit IgG of chromatin extract using sequence specific primers (ERE-A-FP- TGTTCTGTGGCCCAGGTGAGC, ERE-A-RP- AGCTGCTTTTGGGATTCCGTTG, ERE-B-FP- TCGCGGTATGGGTTCTCTCG, ERE-B-RP- TGACCCTTTGTCCTGCACAGC). Each experiment was repeated twice.

### Patient Data Analyses

The datasets analyzed during the current study are available in The Cancer Genome Atlas (TCGA) data portal (https://cancergenome.nih.gov). Gene and miRNA expression data and the corresponding clinical information for the BRCA (1105 samples, TCGA provisional) dataset were obtained through Firebrowse (http://firebrowse.org/) data portal. The normalized reads count for mRNA (Agilent raw data and RSEM normalized) and mature miRNAs (RPM) were downloaded and log2 transformed. From the BRCA dataset, we selected samples with miRNA, mRNA and overall survival (OS) data. The correlations between the expression levels of miRNA and mRNA were calculated using Pearson correlation (GraphPad software). Survival analysis was performed using Kaplan-Meier curve log-rank testing, using Cutoff finder^54^ for best MED1 high- and low-expression selection. Comparison of MED1 and miRNA expression in BC and normal breast was performed using two-tailed Mann Whitney test.

### Cellular assays

The cellular assays were performed as already described in ref.^24^. Briefly, miR-191 or MED1 or PC (control vector) were overexpressed transiently using lipofectamine2000 and 48 hrs post transfection effect on cell survival and cell migration was observed using MTT (3-(4,5-Dimethylthiazol-2-yl)-2,5-Diphenyltetrazolium Bromide) and wound healing assay.

To confirm that miR-191 is responsible for the MED1 mediated effects, MED1 overexpression was coupled with miR-191 inhibition using anti-miR-191/Ctrl oligos and effect on cell survival and migration was looked for using the similar assays as described above. For wound healing assay, the area of migration was estimated by choosing an arbitrary unit scale.

## Electronic supplementary material

Supplementary Information
