# Supplementary material for: Essential role of MED1 in the transcriptional regulation of ER-dependent oncogenic miRNAs in breast cancer
Source: Sci Rep. 2018 Aug 7;8:11805. doi: 10.1038/s41598-018-29546-9 (PMC6081450; doi:10.1038/s41598-018-29546-9)
Supplement: Supplementary file 1 — Supplementary Information [file 41598_2018_29546_MOESM1_ESM.pdf]

Supplementary Information

Essential role of MED1 in the transcriptional regulation of ER-dependent oncogenic miRNAs in breast cancer

Neha Nagpal[1$#], Shivani Sharma[1#], Sourobh Maji[2], Giorgio Durante[3], Manuela Ferracin[3], Jitendra K. Thakur[2*], Ritu Kulshreshtha[1*]

[1]Department of Biochemical Engineering and Biotechnology, Indian Institute of Technology Delhi, New Delhi-110016, India

[2]National Institute of Plant Genome Research, Aruna Asaf Ali Marg, New Delhi-110067, India

[3]Department of Experimental, Diagnostic and Specialty Medicine (DIMES), University of Bologna, 40126 Bologna, Italy
[$]Present Address- Children's Hospital, Harvard Medical School, Boston, MA, USA

[#]Authors contributed equally

* RK and JKT are equivalent corresponding authors.

To whom the correspondence should be addressed: Dr. Ritu Kulshreshtha, Department of Biochemical Engineering and Biotechnology, Indian Institute of Technology Delhi, New Delhi, India-110016 Tel: +91-11-26591017, Fax: +91-11-26582282, email: ritu@dbeb.iitd.ac.in; drritukulshreshtha@gmail.com. Dr. Jitendra K. Thakur, National Institute of Plant Genome Research, Aruna Asaf Ali Marg, New Delhi, India-110067, Tel: +91-11-26735151, email: jthakur@nipgr.ac.in

Running Title: Role of MED1 in regulation of miR-191/425 cluster

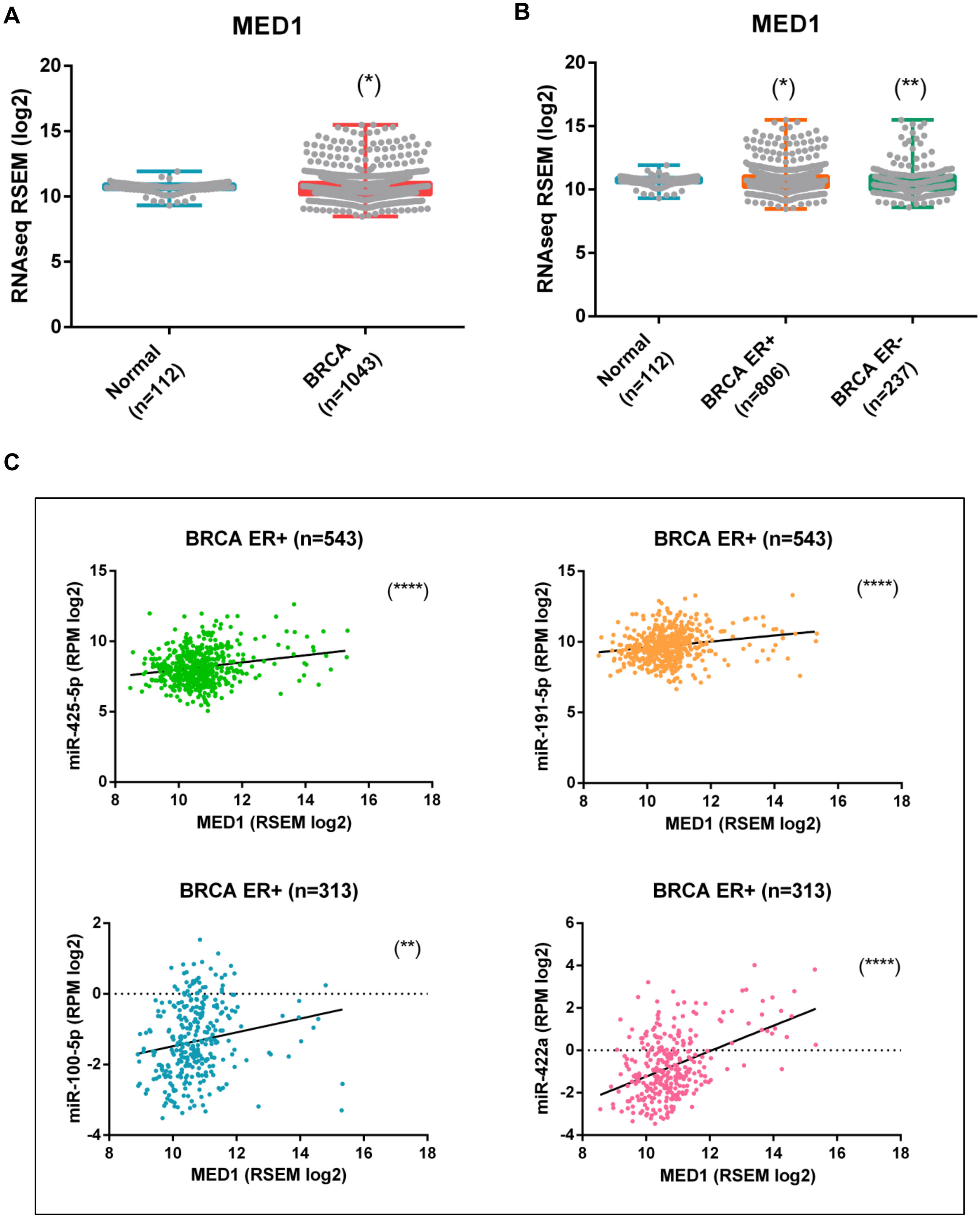
A
MED1
RNAseq RSEM (log2)
(*)
Normal (n=112)
BRCA (n=1043)
B
MED1
RNAseq RSEM (log2)
(*)
(**)
Normal (n=112)
BRCA ER+ (n=806)
BRCA ER- (n=237)
C
BRCA ER+ (n=543)
miR-425-5p (RPM log2)
MED1 (RSEM log2)
(****)
BRCA ER+ (n=543)
miR-191-5p (RPM log2)
MED1 (RSEM log2)
(****)
BRCA ER+ (n=313)
miR-100-5p (RPM log2)
MED1 (RSEM log2)
(**)
BRCA ER+ (n=313)
miR-422a (RPM log2)
MED1 (RSEM log2)
(****)

A

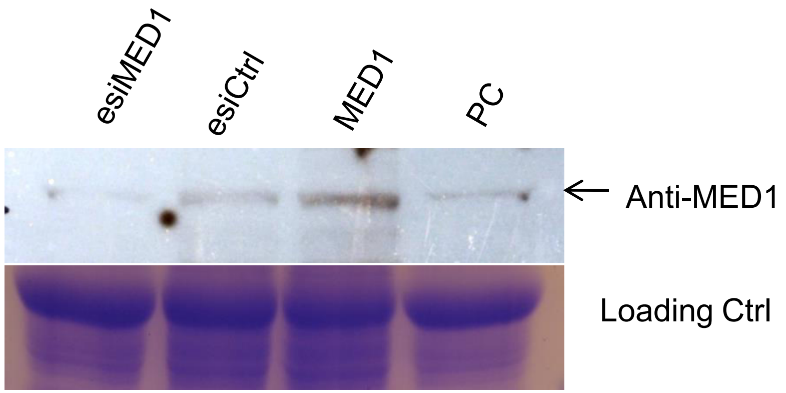
esiMED1
esiCtrl
MED1
PC
Anti-MED1
Loading Ctrl

B

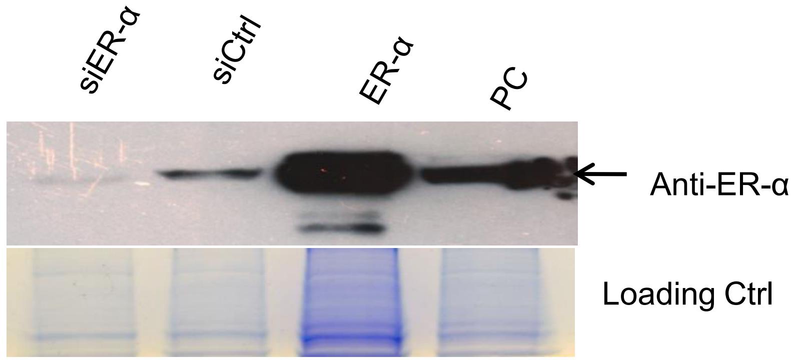
siER-α
siCtrl
ER-α
PC
Anti-ER-α
Loading Ctrl

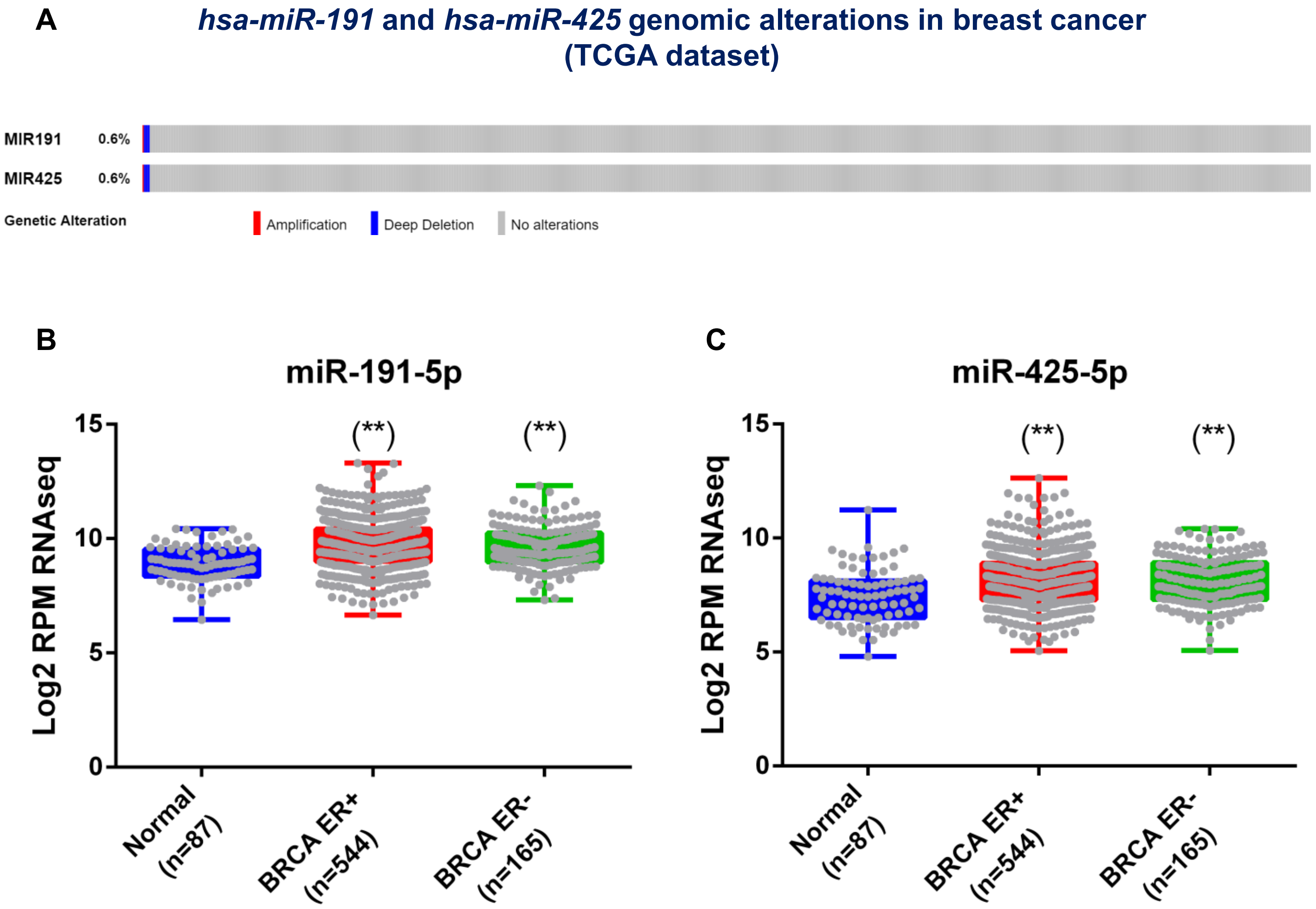
A
hsa-miR-191 and hsa-miR-425 genomic alterations in breast cancer
(TCGA dataset)
MIR191 0.6%
MIR425 0.6%
Genetic Alteration
Amplification
Deep Deletion
No alterations
B
miR-191-5p
Log2 RPM RNAseq
15
10
5
0
(**)
(**)
Normal (n=87)
BRCA ER+ (n=544)
BRCA ER- (n=165)
C
miR-425-5p
Log2 RPM RNAseq
15
10
5
0
(**)
(**)
Normal (n=87)
BRCA ER+ (n=544)
BRCA ER- (n=165)

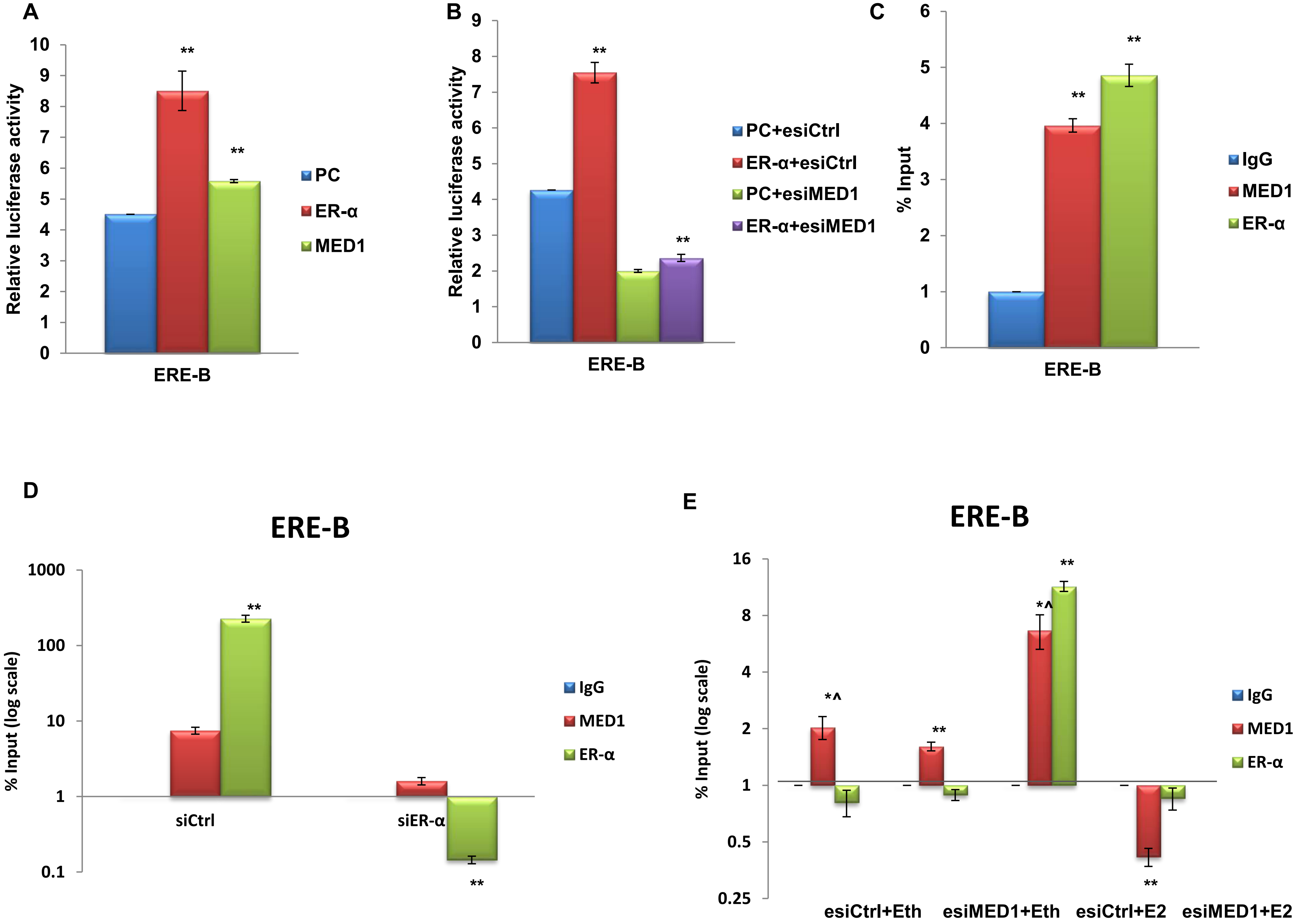
A
Relative luciferase activity
10
9
8
7
6
5
4
3
2
1
0
**
**
PC
ER-α
MED1
ERE-B
B
Relative luciferase activity
9
8
7
6
5
4
3
2
1
0
**
**
PC+esiCtrl
ER-α+esiCtrl
PC+esiMED1
ER-α+esiMED1
ERE-B
C
% Input
6
5
4
3
2
1
0
**
**
IgG
MED1
ER-α
ERE-B
D
ERE-B
% Input (log scale)
1000
100
10
1
0.1
**
**
siCtrl
siER-α
IgG
MED1
ER-α
E
ERE-B
% Input (log scale)
16
8
4
2
1
0.5
0.25
*^
**
*^
**
**
esiCtrl+Eth
esiMED1+Eth
esiCtrl+E2
esiMED1+E2
IgG
MED1
ER-α

Supplementary Data 1 – MED1 expression in breast cancer and correlation between miRNA and MED1 expression.

A. MED1 expression is significantly upregulated in breast cancer, both considering all breast cancer TCGA samples (N=1043) or **B**. analyzing ER+ and ER- patients separately. **C**. Correlation analysis of MED1 levels with miR-191-5p, miR-425-5p, miR-100-5p and miR-422a showing significant positive correlation (Pearson analysis) in ER+ breast cancer patients using TCGA data. P-value was <0.001(**) or <0.00001(****).

Supplementary Data 2–Confirmation of MED1 protein levels after transient overexpression/inhibition.

A. Confirmation of overexpression/ inhibition of MED1 protein levels in MCF7 cells. Level ofMED1 was transiently up/ downregulated by transfecting with pCDNA3.1-MED1 (MED1) or pCDNA3.1 (PC) and esiMED1/esiCtrl. The corresponding effect on MED1 protein levels in responseto MED1 overexpression/downregulation was then checked by western blotting using antibody specific to that of MED1 (Santacruz Biotechnology)**.** **B**. Confirmation of overexpression/ inhibition of ER-α protein levels in MCF7 cells. Level of ER-α was transiently up/ downregulated by transfecting with pCDNA3.1- ER-α (ER-α) or pCDNA3.1 (PC) and si ER-α /siCtrl.

Supplementary Data 3 – Alterations of miR-191-5p and miR-425-5p in breast cancer.

A. miR191/425 is not mutated and rarely amplified in BC (source: TCGA dataset), suggesting a transcriptional regulation of these two miRNAs. The analysis of 796 samples from TCGA miRNA seq experiments demonstrates that both miR-191-5p (**B**) and miR-425-5p (**C**) are significantly upregulated in both ER+ and ER- breast cancer patients compared to normals (p<0.0001 at Mann-Whitney test).

Supplementary Data 4– MED1 and ER-α bind to promoter ERE-B upstream of miR-191/425 cluster.

A. Luciferase activity of ERE-B luciferase construct upstream of miR-191/425 cluster in response toER or MED1 overexpression. **B-E**. Binding of MED1 and ER-α to ERE-B to regulate miR-191/425 cluster. Levels of MED1 were modulated (using esiMED1/esiCtrl) along with overexpression of ER-α and effect on luciferase activity of ERE-B was observed. Increase in luciferase activity observed in response to ER-α (**B**) was not there when combined with the inhibition of MED1. **C**. CHIP assay results showing % input of bound chromatin on ERE-B, element present upstream of miR-191/425 in MCF7 cells using antibody specific to MED1/ ER-α.**D**. CHIP assay result showing recruitment ofboth ER-α and MED1 on ERE-B in response to ER-α inhibition using specific siRNA. **E**. CHIP assay was performed using ER-α or MED1 specific antibodies in response to estrogen/ethanol treatment(E2/Eth (ethanol; vehicle control) 1 x 10^{-8} M for 2 hrs) with/without inhibition of MED1. The graph shows real time PCR based quantification of bound chromatin (% input) on ERE-B in MCF7 cells. (**P<0.05, *^ P<0.1). Error bars denote $\pm$ SD.
